# Genome Analyses of Two Blueberry Pathogens: *Diaporthe* *amygdali* CAA958 and *Diaporthe eres* CBS 160.32

**DOI:** 10.3390/jof8080804

**Published:** 2022-07-29

**Authors:** Sandra Hilário, Micael F. M. Gonçalves, Cátia Fidalgo, Marta Tacão, Artur Alves

**Affiliations:** Centre for Environmental and Marine Studies (CESAM), Department of Biology, University of Aveiro, Campus Universitário de Santiago, 3810-193 Aveiro, Portugal; sandra.hilario@ua.pt (S.H.); mfmg@ua.pt (M.F.M.G.); cifidalgo@ua.pt (C.F.); martat@ua.pt (M.T.)

**Keywords:** CAZymes, *Diaporthe*, effectors, Fusicoccin A, virulence factors, whole genome sequencing

## Abstract

The genus *Diaporthe* includes pathogenic species distributed worldwide and affecting a wide variety of hosts. *Diaporthe amygdali* and *Diaporthe eres* have been found to cause cankers, dieback, or twig blights on economically important crops such as soybean, almond, grapevine, and blueberry. Despite their importance as plant pathogens, the strategies of species of *Diaporthe* to infect host plants are poorly explored. To provide a genomic basis of pathogenicity, the genomes of *D. amygdali* CAA958 and *D. eres* CBS 160.32 were sequenced and analyzed. Cellular transporters involved in the transport of toxins, ions, sugars, effectors, and genes implicated in pathogenicity were detected in both genomes. Hydrolases and oxidoreductases were the most prevalent carbohydrate-active enzymes (CAZymes). However, analyses of the secreted proteins revealed that the secretome of *D. eres* CBS 160.32 is represented by 5.4% of CAZymes, whereas the secreted CAZymes repertoire of *D. amygdali* CAA958 represents 29.1% of all secretomes. Biosynthetic gene clusters (BGCs) encoding compounds related to phytotoxins and mycotoxins were detected in *D. eres* and *D. amygdali* genomes. The core gene clusters of the phytotoxin Fusicoccin A in *D. amygdali* are reported here through a genome-scale assembly. Comparative analyses of the genomes from 11 *Diaporthe* species revealed an average of 874 CAZymes, 101 secondary metabolite BGCs, 1640 secreted proteins per species, and genome sizes ranging from 51.5 to 63.6 Mbp. This study offers insights into the overall features and characteristics of *Diaporthe* genomes. Our findings enrich the knowledge about *D. eres* and *D. amygdali*, which will facilitate further research into the pathogenicity mechanisms of these species.

## 1. Introduction

The intercontinental movement of pathogens along with crop or forestry products can promote the emergence of new pathogens in new ecological niches [1]. However, although the diseases associated with these pathogens may be known, the mechanisms relating to infection biology and pathogenicity/virulence are not entirely understood. In these cases, the sequencing of fungal genomes has been widely implemented by mycologists and plant pathologists [2]. Genome analysis can provide a first attempt to identify genes associated with different pathogenic strategies, understand disease biology, and improve methods and strategies for disease diagnosis [2,3].

The genus *Diaporthe* encompasses species behaving as endophytes, saprobes, and pathogens that play an important role in plant pathology [4]. Currently, more than 300 species supported by DNA sequences are distributed worldwide and have been reported on several hosts, causing diseases in agriculture and forestry [5,6,7]. For example, *Diaporthe eres* (syn. *D. castaneae-mollissimae*) has been reported to cause leaf blight and leaf spot of *Castanea mollissima* [8], *D. eres* (syn. *D. vaccinii*) has been reported to cause twig blight of blueberries [9], and *D. amygdali* is known to cause cankers on almond and peach [10]. Moreover, it is also recognized that the symptoms caused by *D. amygdali* might be associated with the production of a phytotoxin, Fusicoccin A [11].

Studies on *Diaporthe* have been mostly focused on the identification of plant pathogens and endophytes, their pathogenicity [12,13], and their metabolites [14,15]. Although there are 25 published genomes currently available in the NCBI database (https://www.ncbi.nlm.nih.gov/, accessed on 4 May 2022), and 6 genomes deposited in the JGI Portal database (https://genome.jgi.doe.gov/portal/, accessed on 3 May 2022), there is still a lack of in-depth studies on the genomes of species of *Diaporthe*. Nevertheless, recent studies using genomic and transcriptomic approaches have been carried out to understand how species of *Diaporthe* infect their hosts. For instance, Mena et al. [16] revealed insights into the molecular traits involved in the pathogenicity of *D. caulivora* on soybean plants. Gai et al. [17] demonstrated that the genomic analyses of *Diaporthe* species that are responsible for melanose on citrus (*D. citri*, *D. citriasiana*, and *D. citrichinensis*) helped unveil the molecular mechanism of fungicide resistance, pathogen–host interaction, and population genome-related research of this relevant plant pathogen.

Recently, Hilário et al. [18] showed that *D. amygdali* CAA958 was one of the most aggressive species to blueberry plants. Moreover, the ex-type strain of *D. vaccinii* (syn *D. eres*) CBS 160.32 was previously recognized as a threat to blueberry plantations and, thus, was listed as a quarantine organism in Europe [19]. Here, to understand how *Diaporthe* species invade the hosts, we aimed to identify pathogenicity-related genes, candidate effectors, cellular transporters, biosynthetic metabolite gene clusters (BGCs), and carbohydrate-active enzymes (CAZymes) by sequencing and analyzing the genomes of *D. eres* CBS 160.32 and *D. amygdali* CAA958. Additionally, a comparative analysis was performed to gain knowledge on the strategies of *Diaporthe* species to successfully enter and colonize their plant host. For this, we analyzed the genomes of nine important plant pathogens, namely *D. ampelina* DA912, *D. batatas* CRI 302-4, *D. capsici* GY-Z16, *D. caulivora* D57, *D. citri* ZJUD2, *D. citriasiana* ZJUD30, *D. citrichinensis* ZJUD34, *D. helianthi* DHEL01, and *D. longicolla* MSPL 10–6, whose annotations are available and/or published on public databases [16,17,20,21,22,23,24].

## 2. Materials and Methods

### 2.1. Fungal Material and Culture Conditions

*Diaporthe amygdali* strain CAA958 was collected from diseased twigs of *Vaccinium corymbosum* in Portugal [25], stored in a glycerol solution (15%) at −80 °C, and maintained in the culture collection of the Micoteca of University of Minho, hosted at the Center for Biological Engineering, Braga, Portugal. *Diaporthe eres* strain CBS 160.32, previously known as *D. vaccinii* [19], was isolated from *V. macrocarpon* in the USA. It was obtained from the CBS collection of the Westerdijk Fungal Biodiversity Institute, Netherlands. Both strains were cultured on Potato Dextrose Agar medium (Merck, Darmstadt, DE, Germany) at 25 °C for 7 days prior to DNA extraction.

### 2.2. DNA Extraction

Mycelia of *D. amygdali* CAA958 and *D. eres* CBS 160.32 were obtained from cultures grown on 50 mL of fresh Potato Dextrose Broth medium (Merck, Darmstadt, DE, Germany) at 25 °C for 7 days. The mycelium was filtered using sterile filter paper and was ground to a fine powder in liquid nitrogen. DNA was extracted according to Pitcher et al. [26]. The integrity of DNA was assessed using electrophoresis on 0.8% agarose gel and quantified using a Nanodrop 2000 Spectrophotometer (Thermo Fisher Scientific Inc., Waltham, MA, USA).

### 2.3. Genome Sequencing, Assembly, and Prediction

*Diaporthe amygdali* CAA958 and *D. eres* CBS 160.32 genomes were sequenced from 100 ng of genomic DNA by Genome Sequencer Illumina HiSeq (2 × 150 bp paired-end reads) with NovaSeq 6000 S2 PE150 XP (Eurofins, Belgium). Adaptor contamination and low-quality reads were removed from output reads using the Trimmomatic software v.0.39 [27]. The quality of the reads was assessed using the FastQC program [28]. The genome was assembled using SPAdes v.3.14 with kmer size values of 21, 33, 55, and 77 [29]. QUAST web interface [30] was used to assess the quality of the assembly. Assembly completeness was assessed using Benchmarking Universal Single-Copy Orthologs (BUSCO v5.3.2) (https://busco.ezlab.org/, accessed on 5 May 2022). Gene prediction was performed with Augustus v.3.3.3 [31] using *Diaporthe helianthi* gene models as the training set with default parameters.

### 2.4. Dispersed Repeat Sequences and Noncoding tRNA Annotation

Dispersed repeat sequences were masked throughout the genome with the Repeat Masking option (RepeatMasker v.4.0.9) [32] implemented in OmicsBox software v.1.4.12 [33]. Tandem repeat sequences (TRs) were located across the genome using the software Tandem Repeats Finder (TRF) [34]. The tRNAs regions were predicted using the tRNAscan-SE tool with default parameters [35].

### 2.5. Gene Annotation and Functional Analyses

The predicted genes were functionally annotated with OmicsBox software using Blast2Go v.1.2.14 [33] against the NCBI’s nonredundant database, the Kyoto Encyclopedia of Genes and Genomes (KEGG) [36,37,38], and the Gene Ontology (GO) Consortium. The protein sequences were classified based on InterProScan [39] and the Evolutionary Genealogy of Genes: Non-Supervised 105 Orthologous Groups (EggNOG) [40] databases. The analyses were performed using an e-value threshold of 1 × 10^−3^.

The prediction of secreted proteins, including signal peptides, was carried out with SignalP 5.0 [41] with default parameters. Predicted proteins with signal peptides were used to identify putative membrane proteins with DeepTMHMM v.1.0.8 [42], and proteins with no transmembrane structure were selected as secreted proteins. Additionally, EffectorP v.3.0 was used to predict fungal effectors [43]. To identify the proteins involved in pathogenicity, the predicted secretome was used as a query for the BlastP search against the pathogen–host interaction database, with a cut-off e-value set at 1 × 10^−5^ (PHI-database v.4.10), which catalogs verified pathogenicity, virulence, and effector genes from fungal, oomycete, and bacterial pathogens [44]. Biosynthetic gene clusters encoding for secondary metabolites were predicted using the web-based application antiSMASH v.5.0, using the strictness “relaxed” option to detect well-defined clusters and partial clusters with functional parts [45]. Carbohydrate-active enzymes were predicted using the web-based application dbCAN HMMs 5.0 with default settings [46]. Transporters were identified with BlastP analysis against the Transporter Classification Database [47] using an e-value threshold of 1 × 10^−5^. Geneious Prime v.2021.0.3 was used to BlastP against the PHI [44] and Transporter Classification databases [47].

### 2.6. Comparative Analyses

Nine additional fungal genomes were included for comparative analyses (*D. ampelina* DA912, *D. batatas* CRI 302-4, *D. capsici* GY-Z16, *D. caulivora* D57, *D. citri* ZJUD2, *D. citriasiana* ZJUD30, *D. citrichinensis* ZJUD34, *D. helianthi* DHEL01, and *D. longicolla* MSPL 10–6). These genomes, whose annotations are publicly available, were used to perform a comparison of GC content, genome size, BUSCO completeness, predicted proteins, and abundance of CAZymes and BGCs. These taxa include mainly pathogens of citrus [17], grapevine [22], soybean [16,21], sunflower [20], sweet potato [23], and walnut [24].

## 3. Results

### 3.1. Genome Assembly and Genomic Characteristics

Sequencing of *D. amygdali* CAA958 generated more than 23 million reads with 71x-fold genome coverage, while sequencing of *D. eres* CBS 160.32 generated over 24 million reads with approximately 59x coverage. The overall assembly statistics for both genomes are summed up in Table 1. Briefly, the *D. amygdali* CAA958 genome was estimated at 51.5 Mbp with 15,818 predicted coding sequences, from which 55.7% encode for hypothetical proteins (n = 8807). The *D. eres* CBS 160.32 genome size was estimated to be 60.8 Mbp (15.2% larger than *D. amygdali* CAA958) and has 4.1% more predicted coding sequences (n = 16,499) and 13.6% more hypothetical proteins than *D. amygdali* (n = 10,195).

Repetitive sequences are grouped into tandem repeats (TRs) and dispersed repeats (DRs). The total length of DRs in *D. eres* CBS 160.32 and *D. amygdali* CAA958 was estimated at 799,386 bp and 571,940 bp, respectively. Regarding the TRs, 8639 sequences (0.95% of the whole genome) were predicted for *D. amygdali* CAA958, and 33,522 TRs (covering 3.68%) were estimated in the genome of *D. eres* CBS 160.32 (Table 2). Among the predicted tRNAs in the *D. amygdali* CAA958 genome, 8 tRNAs were predicted as possible pseudogenes and 154 as anticodon, while for *D. eres* CBS 160.32, 19 were predicted as possible pseudogenes and 158 as anticodon tRNAs.

### 3.2. Gene Prediction and Functional Annotation

The genome of *D. amygdali* CAA958 was estimated at 15,818 genes and *D. eres* CBS 160.32 at 16,499 genes annotated according to the NCBI’s nonredundant protein, UniProt/Swiss-Prot, EggNOG, KEGG, and GO databases (Tables S1 and S2). From the total 14,012 predicted proteins in *D. amygdali* CAA958, signal peptides were identified in 1874 (13.4%) proteins (Table S3), and transmembrane structures were detected in 3122 (19.7%) proteins (Table S4). From those proteins with a signal peptide and no transmembrane structure, 1562 (31.7%) were predicted as secreted proteins (secretome) (Table S5). In the genome of *D. eres* CBS 160.32, 14,625 (88.6%) proteins were predicted, from which 1806 (12.3%) had a signal peptide, 3220 (22.0%) had a transmembrane structure, and 1616 (11.1%) were predicted as secreted proteins.

**Figure 1:**
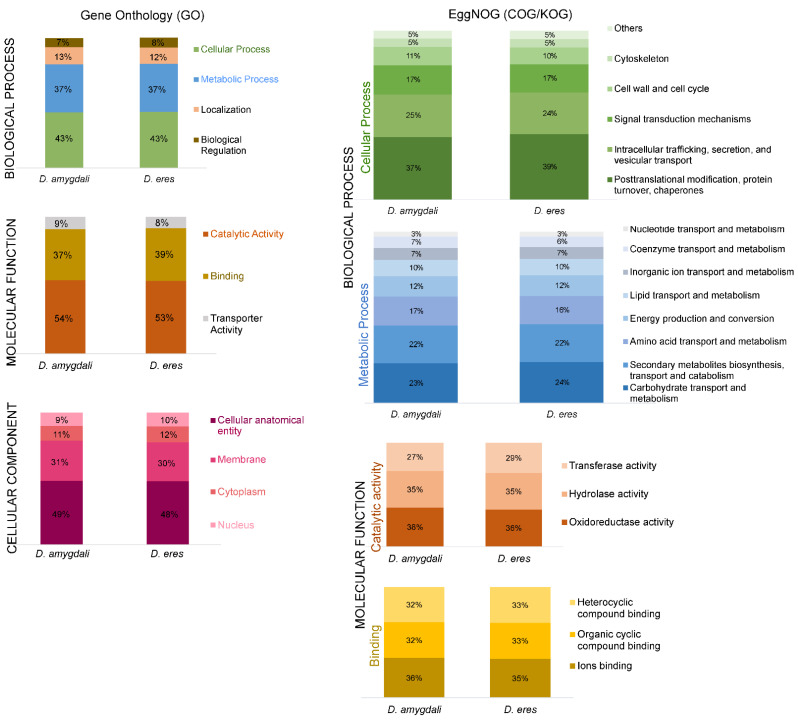
Gene ontology (GO; **left** panel) and EggNOG functional annotation (**right** panel) of *Diaporthe amygdali* CAA958 and *Diaporthe eres* CBS 160.32.

Functional analysis (GO, Biological Process) of *D. amygdali* CAA958 (Table S1) and *D. eres* CBS 160.32 (Table S2) revealed that most genes are involved in cellular and metabolic processes, localization (establishment of cellular component location), and biological regulation (Figure 1). In both genomes, the genes included in the cellular process category were mostly classified as chaperones or participants in post-translational modification or protein turnover, intracellular trafficking, secretion, and vesicular transport, signal transduction mechanisms, cell wall and cell cycle control, cytoskeleton, and others, which include defense mechanisms, nuclear and extracellular structure, and cell motility. Regarding the metabolic process category, genes of both species are largely involved in the transport and metabolism of carbohydrates, biosynthesis, transport and catabolism of secondary metabolites, amino acids, energy production and conversion, and metabolism of lipids and inorganic ions. In GO, Molecular Functions, genes are mostly involved in catalytic activity, binding, and transporter activity. Within the catalytic activity, genes are classified as participating in oxidoreductase, hydrolase, and transferase. Analyses of Cellular Component (GO) show that most genes are involved in the cellular anatomical entity, membrane, cytoplasm, and nucleus.

### 3.3. Fusicoccin A Biosynthesis

**Figure 2:**
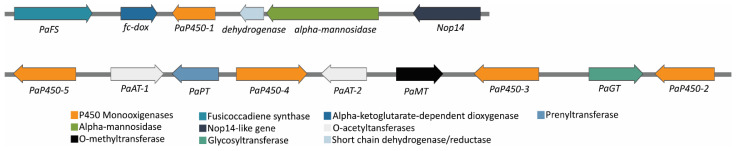
Fusicoccin A biosynthetic gene clusters found in *Diaporthe amygdali* CAA958. Gray horizontal lines represent genomic sequences. Color-coded arrows represent the predictive function of different genes. Arrows indicate the direction of transcription of the gene.

Fusicoccin A is a phytotoxin produced by *D. amygdali*. Here, the genes involved in its biosynthesis were identified at two different loci. The core gene clusters for Fusicoccin A comprise 13 genes that were found only in the genome of *D. amygdali* CAA958. The clusters include a *PaFS* fusicoccadiene synthase, five cytochrome P450s (*PaP450-1*, *PaP450-2*, *PaP450-3*, *PaP450-4*, *PaP450-5*), two O-acetyltransferases (*PaAT-1*, *PaAT-2*), a methyltransferase (*PaMT*), a prenyltransferase (*PaPT*), an alpha-ketoglutarate dependent dioxygenase, a glycosyltransferase (*PaGT*), and a short-chain dehydrogenase/reductase (Figure 2). The schematic organization of the loci was created using the free software SnapGene Viewer 4.0.1 (https://www.snapgene.com/, accessed on 12 November 2021).

### 3.4. Virulence Factors, Effectors, and Strategies to Overcome Host Responses

From the predicted secretome of *D. eres* CBS 160.32, we identified 88 genes encoding for CAZymes (5.4%), and 458 secreted proteins were identified in the pathogen–host interaction (PHI) database [44] (28.1%) (Table S6). Of these secreted proteins, 98 were identified as effector candidates (20%), including carboxylesterases, lipases, peptidases, glycosyl hydrolases, and several hypothetical proteins (Table S7). In the secretome of *D. amygdali* CAA958, we identified 454 genes encoding for CAZymes (29.1%), 469 in the PHI-database (29%) (Table S6), and 109 effectors (5.6%) such as pectate lyases, cellulases, endopolygalacturanases, cutinases, and laccases (Table S7). Some predicted effectors were also found on both *D. eres* and *D. amygdali* genomes, including the CFEM domain (Common in Fungal Extracellular Membrane), necrosis, and ethylene-inducing peptide 1 (Nep1)-like proteins (NLP), metalloproteases, pectinesterases, and acetylxylan esterases. Moreover, genes encoding for proteins with potential roles in pathogenesis, such as the velvet complex, virulence protein sorting, and proteases, were also identified on both genomes (Table 3). Genes encoding proteins that are produced to overcome immune plant defenses were also detected on both genomes analyzed, such as genes encoding arylsulfatases, salicylate hydroxylase, tyrosinase, homogentisate dioxygenase (HGD), fumarylacetoacetate hydrolase (FMH), cytochrome P450 monooxygenases superfamily, and flavin-containing monooxygenases.

### 3.5. Cellular Transporters

All transporter classes (TC) were detected in our analyses: TC 1–9 (Table 4). A total of 2325 and 2238 genes encoding for transporters were identified (Table S8) in *D. amygdali* CAA958 and *D. eres* CBS 160.32, respectively, accounting for 14.7% and 13.6% of the total predicted genes. Overall, the electrochemical potential-driven transporters (TC 2) were the most prominent group, and in both genomes represent an average of 42% of the annotated transporters, followed by the primary active transporters (TC 3, average = 17%) and channels and pores (TC 1, average = 16%). The TC 2 was the largest category of cellular transporters identified in *D. eres* CBS 160.32 and *D. amygdali* CAA958 (Table 4). Both genomes encode transporters involved in the transport of zinc (e.g., *zrt1*, *zrt2*, *zrt3*), sulfur (e.g., *mup1*, *mup3*), siderophores (e.g., *mirB*), and MFS transporters (major facilitator superfamily) such as sugar/H+ symporter (e.g., *stl1*), glucose/xylose symporter, inositol, and glycerol transporters. On both genomes, we also detected genes encoding for ABC transporters (TC 3) that confer antifungal resistance to fluconazole (e.g., *fcr1*) and voriconazole (e.g., *atrF*). Some accessory factors involved in transport (TC 8) were found on *D. eres* and *D. amygdali* genomes, such as genes encoding tetraspanin (e.g., *pls1* and *tsp3*) and peroxiredoxins (e.g., *prx1*) transporters.

### 3.6. Comparative Analyses

#### 3.6.1. Predicted Genes and Genome Statistics

Most published genomes of *Diaporthe* do not have available functional annotations; thus, we chose *D. ampelina* DA912, *D. batatas* CRI 302-4, *D. capsici* GY-Z16, *D. caulivora* D57, *D. citri* ZJUD2, *D. citriasiana* ZJUD30, *D. citrichinensis* ZJUD34, *D. helianthi* DHEL01, and *D. longicolla* MSPL 10–6 to perform a comparative analysis. These species were chosen mainly due to their importance as plant pathogens and their annotation’s availability on public databases and published works. Overall, the genomic features vary among the analyzed species regarding their GC content, genome size, and BUSCO completeness. The GC content ranges from 43.9% to 52.9%, in which *D. helianthi* DHEL01 displayed 17% less GC content (43.9%) than *D. caulivora* D57 (52.9%). The number of predicted genes ranged from 10,704 (*D. ampelina* DA 912) to 18,385 (*D. caulivora* DS7), in which *D. ampelina* has 41.8% fewer genes than *D. caulivora*. The size of the genomes had an average of 57.1 Mbp per species, ranging from 51.5 Mbp in *D. amygdali* CAA958 to 63.6 Mbp in *D. helianthi* DHEL01, being the genome of *D. amygdali* 19% smaller than *D. helianthi* (Table 5). A near completeness of the assemblies was also verified by BUSCO analyses, which reported an average of 98.4% completeness among all species analyzed. The number of secreted proteins showed an average of 1640 per species, ranging from 2043 in *D. citrichinensis* ZJUD34 to 1224 in *D. batatas* CRI 302-4.

#### 3.6.2. CAZymes

A total of 857 and 859 genes encoding for putative CAZymes were identified in the *D. amygdali* and *D. eres* genomes, respectively (Table 6, Tables S9 and S10). The glycoside hydrolases (GH) are by far the largest family of CAZYmes in these genomes. About 404 and 398 protein-coding genes belonging to more than 65 different glycoside hydrolases made up approximately 47% of *D. amygdali* and 46% of *D. eres* cell-wall degrading repertoire, respectively. The main GH subfamilies detected on both *D. amygdali* and *D. eres* genomes were β-glucosidases (GH3), endo-β-1,4-glucanases/cellulases (GH5), α-amylases (GH13), xyloglucan transglucosylases (GH16), chitinases (GH18), polygalacturonases (GH28), and β-xylosidase/α-L-arabinofuranosidases (GH43). Regarding glycosyltransferases (GT), GT1 (uridine diphosphate UDP-glycosyltransferase) and GT2 (cellulose/chitin synthase) were the most abundant. Carbohydrate-binding modules (CBM) families involved in starch-binding (CBM20) and L-rhamnose-binding (CBM67) were also the most abundant in both genomes analyzed. Among the auxiliary activity (AAs) family, cellobiose dehydrogenases (AA3), xylo- and cello-oligosaccharide oxidases (AA7), and copper-dependent lytic polysaccharide monooxygenases (AA9) were the most predominant. Regarding the carbohydrate-esterases (CE) subfamilies, acetylxylan esterases (CE1) and cutinases (CE5) were the most abundant, while the pectase lyases PL1 and PL3 (polysaccharide lyases) were also the most prominent families on *D. amygdali* and *D. eres* genomes.

**Figure 3:**
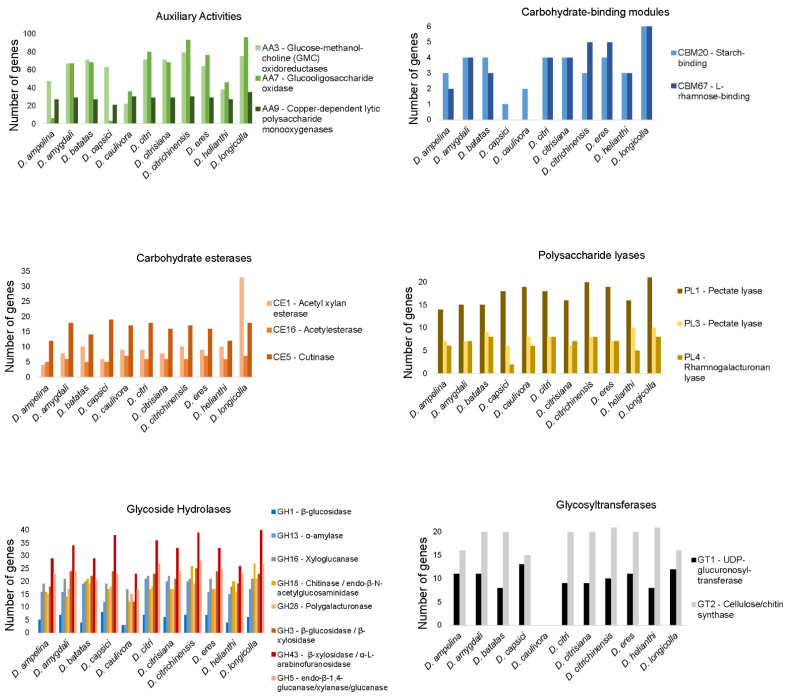
Number of predicted genes encoding for the most abundant carbohydrate-active enzyme families in all genomes of the analyzed *Diaporthe* species.

Overall, the total number of CAZymes per species was 874, ranging from 696 in *D. ampelina* to 1221 in *D. longicolla*. Although all classes of CAZymes were detected, glycoside hydrolases and auxiliary activities were the two groups with the most predicted proteins. Families AA3, AA7, AA9, CBM20, CBM67, CE1, CE16, CE5, PL1, PL3, PL4, GH1, GH13, GH16, GH18, GH43, GT1, and GT2 were the most abundant among all species analyzed (Figure 3). Overall, *D. longicolla* displayed the highest CAZyme content, including the families AA7, CBM20, CBM67, CE1, PL1, and GH43, followed by *D. citrichinensis* with an abundance of AA7, CBM67, PL1, GH18, and GH28 families. Contrarily, the GT1 family was most predominant in *D. capsici* and *D. longicolla* genomes.

#### 3.6.3. BGCs

There are 86 and 88 BGCs involved in the secondary metabolism of *D. amygdali* and *D. eres*, respectively (Table S11). The BGCs identified on the *D. amygdali* genome encode 10 terpenes, 3 indoles, 36 t1PKs (type 1 polyketide synthases), 10 NRPS (nonribosomal peptide synthase), 6 t1PKs-NRPS, 11 NRPS-like, 2 t1PKs-indole, and one of each: siderophore, Fungal-RiPP, t1PKS-NRPS-indole, NRPS-NRPS-like, t1PKs-t3PKs, t3PKs, “other,” and other-t1PKs. Of these 86 BGCs, clusters 1, 21, and 24 have 100% similarity with known BGCs, such as fusarin (mycotoxin), clavaric acid (anticancer), and alternariol (phytotoxic and antifungal), respectively. Clusters 2, 12, 24, and 41 showed homologies with fusarielin H (antifungal) (25%), betaenone (37%), alternapyrone (40%) (phytotoxins), and squalestatin (antifungal) (40%).

In *D. eres*, the 88 BGCs identified encode for 8 terpenes, 5 indoles, 35 t1PKs (type 1 polyketide synthases), 10 NRPS (nonribosomal peptide synthase), 6 t1PKs-NRPS, 12 NRPS-like, 2 terpene-NRPS-like, 2 t1PKs-NRPS-like, 2 t1PKs-NRPS-indole, and one of each: siderophore, Fungal-RiPP, NRPS-like-indole, t1PKS-terpene, t1PKs-indole, and “other.” From the 88 secondary metabolite gene clusters identified, clusters 179, 197, and 297 have 100% similarity with known BGCs, such as the phytotoxins alternariol, mullein, and ACT toxin II, respectively. Clusters 271, 268, and 134 were also found to have homologies with the phytotoxins alternapyrone (40%) and cercosporin (31%) and with PR toxin (mycotoxin) (50%), respectively.

Moreover, other BGCs encoding for betaenone, cercosporin, and PR toxin were detected among the genomes analyzed, but with a similarity ranging from 22% to 60%, indicating that some genes may be truncated. The *Diaporthe eres* cluster 268 showed a gene similarity of 31% with the cercosporin cluster, and it contains only three genes responsible for the biosynthesis of this compound: *ctb3* (cercosporin toxin biosynthesis protein), *ctb1* (PKS), and *ctb2* (O-methyltransferase). Cluster 12 from *D. amygdali* and cluster 360 from *D. eres* showed 37% similarity with the betaenone BGCs and contains an HR-PKS, an enoyl reductase, a short-chain dehydrogenase reductase, and a cytochrome P450 but lacks a dehydrogenase and a FAD-dependent oxidase. Cluster 271 of *D. eres* contains genes involved in PR toxin biosynthesis: a terpene cyclase, an aristolochene synthase, an oxidoreductase, an oxidase, two P450 monooxygenases, a transferase, and two dehydrogenase enzymes.

**Figure 4:**
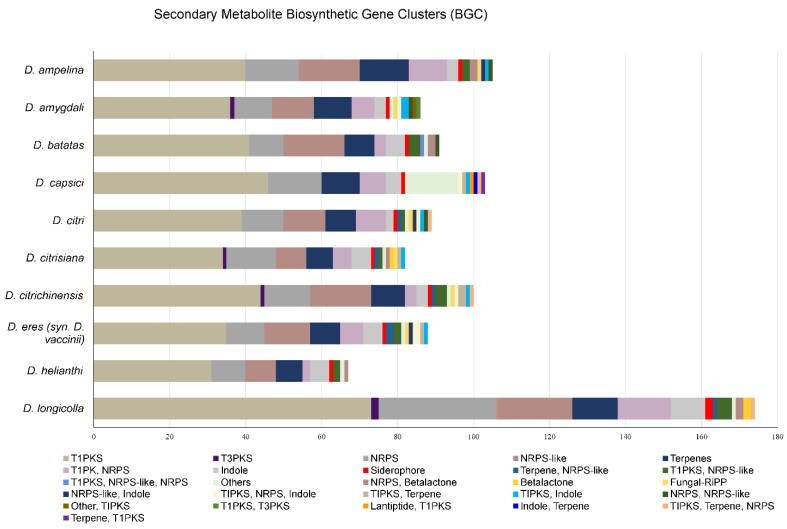
Biosynthetic gene clusters (BGCs) identified in the genomes of *Diaporthe* analyzed. *Diaporthe caulivora* BGCs are not included because the genome is not publicly available.

The genomes analyzed were rich in gene clusters that are involved in the synthesis of secondary metabolites. *Diaporthe longicolla* contains the highest number of BGCs (n = 174), and *D. helianthi* has the lowest (n = 67). *Diaporthe amygdali* and *D. eres* contain 38.5% and 49.4% fewer BGCs than *D. longicolla*, respectively. Overall, all species were estimated with an average of 101 BGCs per species. Type 1 polyketide synthases were the most abundant type of gene clusters, followed by NRPS, NRPS-like, terpenes, and t1PKs-NRPS (Figure 4). Several BGCs encoding phytotoxins with 100% similarity with known BGCs were detected in the genomes analyzed. For example, fusarin BGC was found on *D. batatas*, *D. helianthi*, *D. longicolla*, and *D. amygdali*; alternariol BGC was detected in *D. amygdali*, *D. destruens*, *D. eres*, and *D. capsici*; and mellein BGC was also found in *D. eres*, *D. capsici*, *D. destruens*, and *D. longicolla*. It is worth noting that the ACT toxin BGC was detected only in *D. batatas*, *D. capsici*, *D. citrisiana*, and *D. eres*.

## 4. Discussion

The genomes of *D. amygdali* CAA958 and *D. eres* CBS 160.32 were sequenced, analyzed, and compared with the genomes of *D. ampelina* DA912, *D. batatas* CRI 302-4, *D. capsici* GY-Z16, *D. caulivora* D57, *D. citri* ZJUD2, *D. citriasiana* ZJUD30, *D. citrichinensis* ZJUD34, *D. helianthi* DHEL01, and *D. longicolla* MSPL 10–6 in order to understand the main strategies that *Diaporthe* species use to infect and colonize their hosts.

While plants develop defense mechanisms against fungal pathogens, fungi develop strategies to attack their hosts and manipulate plant immune responses [63]. Effectors such as the CFEM domain, NEP1-like protein, and metalloproteases, which are known to manipulate the host’s hypersensitive response, acting as toxins to induce plant cell death, thereby favoring early pathogen colonization, were detected on both *D. amygdali* CAA958 and *D. eres* CBS 160.32 genomes [64]. Pathogenicity genes were also detected in both genomes, including vacuole protein sorting (which enhances stress resistance to survive within the host [62]), virulence protein SSD1 (involved in tolerance to host immune response [61]), and subtilisin-like serine protease (participates in the degradation of pathogenesis-related proteins produced by the host [57]).

Fungi can quickly adapt to changing environments and develop strategies to overcome immune plant defenses due to their genetic flexibility [65]. To overcome severe nutrient limitations imposed by the host (e.g., sulfur, which is an essential element required for the growth and function of all fungal cells [66,67]), fungi exhibit responses to alleviate the nutrient deficiency [66]. This includes transport systems (e.g., sulfate and methionine permease) allowing the uptake of the sulfate produced and sulfate starvation-induced (SSI) proteins (e.g., arylsulfatase) involved in sulfur scavenging from the environment [68]. Given that genes encoding for sulfur transporters and arylsulfatases were identified in the genomes of *D. amygdali* and *D. eres*, it is suggested that both species may take advantage of sulfur from their hosts for a successful infection, thus ensuring fungal survival in the host’s microenvironment. Moreover, genes encoding for salicylate hydroxylase, tyrosinase, homogentisate dioxygenase (HGD), and fumarylacetoacetate hydrolase (FMH) [69,70] were also identified. The presence of these genes supports the hypothesis that *D. eres* and *D. amygdali* may be able to degrade salicylic acid and plant phenylpropanoid precursors, which are produced by the host as defense mechanisms [71]. Genes encoding for the biosynthesis of carbohydrates inositol (e.g., inositol 5-phosphatase) and mannitol (e.g., mannitol-1-phosphate 5-dehydrogenase) were also detected, involved in the inositol phosphate metabolism and fructose and mannose metabolism pathways, respectively. In fungi, inositol plays a major role in metabolic adaptation, fungal virulence, and sexual development [72]. Reynolds [73] reported the importance of inositol acquisition in the biology and pathogenesis of some fungal pathogens (e.g., *Candida albicans*). Moreover, it is reported that some fungal plant pathogens use mannitol to detoxify reactive oxygen species (ROS) produced by plants [74]. This suggests that both *D. amygdali* and *D. eres* may take advantage of inositol to proliferate and cause infection in their hosts and mannitol to counteract ROS-mediated defenses produced by the host.

A high number of cellular transporters were annotated on both *Diaporthe* species, suggesting the ability to transport molecules to enhance pathogenicity, secondary metabolites, and sugars into the cell [75]. The access to sugars that are released from complex plant polysaccharides relies on the ability of fungi to secrete a large number of sugar transporters from the MFS transporters (TC.2) [76]. In fact, TC 2 was the largest category identified in *D. eres* and *D. amygdali*. Sugar/H+ symporter, glucose/xylose symporter, inositol, and glycerol transporters can recognize and transport more than one type of sugar, such as xylose, glucose, and cellobiose, into the cell [77]. The high number of annotated transporters detected is corroborated by studies on other fungi colonizing plants, such as *Botryosphaeriaceae*, which ranges from 3143 in *Dothiorella sarmentorum* to 2185 in *D. iberica* [78]. In addition to the transport of sugars, peroxiredoxin and tetraspanin transporters were also detected. Rocha et al. [79] suggested that peroxiredoxin plays an important role in the development and pathogenicity of *Aspergillus fumigatus* and *M. oryzae*. Jimenez-Jimenez et al. [80] also stated that tetraspanins are crucial for appressorium-mediated penetration into the host and act as coordinators of the infection process of *M. oryzae* and *Botrytis cinerea*. Therefore, we suggest that *D. eres* CBS 160.32 and *D. amygdali* CAA958 display traits in the genomes to support virulence and persistence into the hosts.

Fusicoccin A is a diterpene glucoside, discovered in 1964 as a fungal phytotoxin, produced by *Fusicoccum amygdali* (syn. *D. amygdali*) [81], whose structure was characterized in 1968 [82]. From a draft genome of the *Phomopsis amygdali* (syn. *D. amygdali*) strain Niigata-2, Noike et al. [83] suggested that Fusicoccin biosynthetic genes are located at two different loci: one containing four genes and the other nine genes. In fact, our results showed that these two gene clusters involved in the biosynthesis of Fusicoccin are composed of 13 genes and were detected in *D. amygdali* CAA958. Fusicoccin A is known to induce irreversible opening of stomata, causing uncontrolled transpiration, leading to the development of cankers on branches, as well as the chlorosis and necrosis of distal leaves in almonds and peach trees [11,82]. When tested on stems/twigs or detached leaves, Fusicoccin can also cause the stomatal opening in a wide range of plants such as: tobacco (*Nicotiana tabacum*), sorghum (*Sorghum bicolor*), cucumber (*Cucumis sativa*), and lucerne (*Medicago sativa*) [81,84]. It is interesting to note that some studies have outlined that Fusicoccin may also contribute to improving plant physiological performance [11]. For instance, some biological activities are attributed to this diterpene glucoside such as the induction of abscission [85], potassium uptake [86], cell enlargement, or stimulation of seed germination [87]. Thus, this suggests that besides its reported phytotoxicity, Fusicoccin can also be used as a plant growth regulator in agriculture, as well as a biochemical agent for plant physiology [88].

The results hinted at a possible correlation between the number of genes and the genome size of the *Diaporthe* species considered in this study. Most fungal species with available genomes exhibit a genome size that ranges from 30 Mbp to 40 Mbp (average = 37.2 Mbp), while genome sizes of sequenced plant pathogenic ascomycetes are slightly larger (average = 39.4 Mbp) [2,78]. However, our results show a relatively large genome size among all *Diaporthe* species analyzed, falling within the 51.5–63.6 Mbp range, with an average of 57.1 Mbp. Although still poorly understood, some studies have revealed that large genome sizes and high numbers of genes are common in fungal plant pathogens [89]. This outcome might be explained by the presence of genes related to host colonization traits (e.g., production of CAZymes, peroxidases) that are under high selective pressure, thus resulting in gene duplication events that play important roles in fungal evolution and adaptation [78,90].

For a successful infection, pathogens may need to break the plant cell wall, which is composed of polysaccharides such as cellulose, beta-glucans, hemicellulose, and pectin [22,91]. Plant pathogens display a wide variety of CAZymes that are involved in the degradation of these plant polysaccharides [92]. All genomes analyzed in this study had abundant numbers of CAZymes (an average of 874 per species), GHs being the most predominant. GHs functions of β-glucosidases, β-xylosidases, amylases, glucanases, L-arabinofuranosidase, and endo-β-1,4-cellulases were present in all *Diaporthe* species used in this study, thus playing an important role in fungal pathogenicity. For instance, endo-β-1,4-cellulases is one of the families commonly found in *Neofusicoccum parvum* [22], while pectinases, hemicellulases, and cellulases secreted by *Valsa mali* are crucial in apple infection [56]. In the same way as the GH, AA functions such as cellobiose dehydrogenase and gluco-oligosaccharide oxidase, which assist lignocellulolytic enzymes in the degradation of plant biomass, were also abundant among all *Diaporthe* species analyzed. Moreover, CBMs that bind to glycoside hydrolases to enhance plant cell wall degradation (e.g., L-rhamnose-binding and starch-binding) and PLs degrading glycosaminoglycans and pectin were also detected among all species analyzed. Our results corroborate previous studies in which the capacity of plant cell wall degradation is linked to fungal lifestyle (necrotrophic, hemibiotrophic, and biotrophic) [93,94]. For instance, some authors have documented that higher numbers of hydrolytic enzymes are most prominent in hemibiotrophs (e.g., *M. oryzae*, *D. longicolla*, *N. parvum*), necrotrophs (e.g., *B. cinerea*), saprobes (e.g., *Paraphaeosphaeria sporulosa*), and endophytes/latent pathogens (e.g., *Periconia macrospinosa*) than in biotrophs [21,78,93]. Therefore, given that species of *Diaporthe* are hemibiotrophs [95], it is expected that all species analyzed exhibit a high number of CAZymes.

The proportion of CAZymes in the *D. amygdali* secretome (29.1%) is consistent with previous reports in other *Diaporthe* species, such as *D. capsici* and *D. citri* (30.7%), *D. caulivora* (30.6%), and *D. longicolla* (31.4%), emphasizing the importance of CAZymes in *Diaporthe* pathogenicity [16]. Nevertheless, the proportion of CAZymes in the secretome of *D. eres* CBS 160.32 was estimated at 5.4%, which is low when compared with the abovementioned species (average = 30.5%). The relatively low number of secreted hydrolytic enzymes may explain the lack of pathogenicity of *D. eres* CBS 160.32, which is consistent with studies that categorize this species as a weak pathogen on blueberry plants [18,96].

Moreover, hemibiotrophic plant pathogens usually possess higher numbers of genes involved in the biosynthesis of secondary metabolites than biotrophic pathogens [97]. In fact, an abundance of BGCs was found in all species analyzed. These secondary metabolites can be toxic polyketides, nonribosomal peptides, terpenes, and indoles that induce plant cell death and lead to disease development [64]. The genomes of *Diaporthe* analyzed contained many BGCs, especially t1PKS, NRPS, NRPS-like, and terpenes. Although the products of some clusters are unknown, some of them could be determined. These compounds included phytotoxins (alternariol, mellein, ACT toxin II) and mycotoxins (fusarin). Although ACT toxin is recognized as a host-specific toxin from citrus infecting *A. alternata* [98], it is worth noting that the ACT toxin-producing gene cluster was detected in *D. eres* and *D. capsici*. However, the ACT toxin II BGC has also been detected in species of *Botryosphaeriaceae* [64]. Therefore, it is suggested that the genes from the cluster responsible for the biosynthesis of the ACT toxin may have been acquired from horizontal gene transfer, as previously suggested by Wang et al. [99].

## 5. Conclusions

This study represents the first report of the *Diaporthe amygdali* and *D. eres* genome sequence. Pathogenicity factors, effectors, cellular transporters of sugars and ions, phytotoxins, and CAZymes were identified on both genomes. The number of CAZymes identified in the secretome of *D. eres* CBS 160.32 suggests that hydrolytic enzymes may not be the most relevant mechanism adopted by this species as a strategy to infect plant hosts. The comparative genome analyses revealed that species of *Diaporthe* exhibit great diversity in the number of hydrolases, transferases, lyases, and oxidoreductases. These are responsible for the breakdown or modification of plant cell wall polysaccharides, suggesting the ability to surpass the plant cell wall. The high number of predicted CAZymes may reflect an ecological selection and adaptation of these fungi to efficiently degrade the available biomass as a carbon source. The genomic data of *D. amygdali* CAA958 and *D. eres* CBS 160.32 will add valuable information for further research into the mechanisms of *Diaporthe* that are involved in pathogenicity. Plant pathogen genomes alone are not sufficient to unravel pathogen–host interactions. Therefore, future studies using Dual RNA sequencing (RNA-Seq) technology, which allows for the analyses of both host and pathogen transcriptomes, may provide better insight into the biology of pathogen infection, as well as host defense mechanisms.

## Figures and Tables

**Table 1:** Genome assembly overview and gene statistics for *Diaporthe amygdali* CAA958 and *Diaporthe eres* CBS 160.32.

Genome Features	*D. amygdali*	*D. eres*
Genome assembled	51.5 Mbp	60.8 Mbp
Number of contigs (>500 bp)	267	2524
Largest contig length	4,327,563 bp	1,105,552 bp
N50 contig length	1,008,325 bp	169,851 bp
N75 contig length	622,097 bp	74,774 bp
GC content	52.1%	47.6%
BUSCO * completeness	98.3%	98.4%
Predicted genes	15,818	16,499
Predicted proteins with signal peptides	1874	1806
Secreted proteins	1562	1616
Candidate effectors	109	98
Total length of coding genes	23,649,268 bp	24,024,391
Average length of predicted genes	1495 bp	1456 bp
Total length of predicted genes/Genome assembled	45.9%	39.5%
Average number of exons per gene	3	3
Average number of introns per gene	2	2

* BUSCO, Benchmarking Universal Single-Copy Orthologs.

**Table 2:** Statistical results for repetitive sequences, tandem repeats, and tRNAs in *Diaporthe amygdali* CAA958 and *Diaporthe eres* CBS 160.32.

Type ^a^		*D. amygdali* CAA958			*D. eres* CBS 160.32		
		Number	Total Length (bp)	Genome Content (%)	Number	Total Length (bp)	Genome Content (%)
Interspersed and terminal repeats	LTRs	131	12,096	0.0235	140	12,642	0.0208
	DNA transposons	174	9942	0.0193	143	11,693	0.0192
	LINEs	18	1207	0.0023	21	1611	0.0027
	SINEs	0	0	0	0	0	0
	Rolling circles	4	243	0.0005	0	0	0
	Small RNA	55	8582	0.0167	52	8523	0.0140
	Satellites	25	1927	0.0037	18	1434	0.0024
	Simple repeats	11,895	486,262	0.9445	15,264	686,233	1.1289
	Low complexity	1067	51,681	0.1004	1571	77,250	0.1271
	TOTAL	13,369	571,940	1.1109	17,209	799,386	1.3151
Tandem repeats		8639	478,007	0.9459	33,522	2,237,060	3.6802
tRNAs		162	15,038	0.0292	177	17,216	0.0283

**^a^** LTRs, long terminal repeats; LINEs, long interspersed nuclear elements; SINEs, short interspersed nuclear elements.

**Table 3:** Putative proteins involved in fungal pathogenesis identified in the genomes of *Diaporthe amygdali* CAA958 and *Diaporthe eres* CBS 160.32.

Putative Protein	*D. amygdali* CAA958	*D. eres* CBS 160.32	Function	References
Acid aspartase	√	×	Role in the mechanisms of virulence during fungal infection, participating in the degradation of the host’s physical barriers	[48]
Aminobutyrate aminotransferase	√	√	Metabolization of γ-aminobutyric acid, providing pathogen nitrogen requirements during infection	[49]
Aminopeptidase, carboxypeptidase	√	√	Protease required by fungi for host peptide degradation during pathogenesis	[50]
Cerato-ulmi	√	×	Hydrophobic proteins secreted by filamentous fungi (*Ophiostoma* species). It possesses properties of a wilt toxin in susceptible elms, such as *Ulmus americana*	[51,52]
Chitin synthases	√	√	Enzymes that serve as a pathogen-associated molecular pattern (PAMP), triggering immune responses in host plants. Reported in *Magnaporthe oryzae*, *Botrytis cinerea*, *Fusarium graminearum*, and *F. verticillioides*	[53]
Metalloprotease	√	√	Zinc-chelating protease that plays an essential role in microbial pathogenesis. In *M. oryzae*, it is an effector that triggers host defense response	[54]
Nudix proteins	√	√	Important virulence components manipulating host defense mechanisms	[55]
Siderophores	√	√	Chelators synthesized to be involved in iron uptake, intracellular transport, and storage. Essential virulence factors allow the fungus to overcome severe iron limitation imposed by the host	[56]
Subtilisin-like serine protease	√	√	Proteases that are released in infected plant host to degrade pathogenesis-related proteins and disrupt host cell membranes	[57]
Tripeptidyl-peptidase	√	×	Acidification of the microenvironment in the host facilitates the proliferation of the pathogen	[58]
Velvet proteins	√	√	Promotion of chromatin accessibility and expression of biosynthetic gene clusters involved in pathogenicity as mycotoxins, pigments, and hormones	[59]
Virulence protein SSD1	√	√	Important for *M. grisea* to colonize rice leaves, leading to evasion and tolerance of the host immune response	[60,61]
Vacuole protein sorting	√	√	Proteins involved in the delivery of soluble vacuolar compounds, metabolite storage, and osmoregulation. Essential for fungal growth and pathogenesis	[62]

**Table 4:** Number of genes predicted to code for transporters in the genomes of *Diaporthe amygdali* CAA958 and *Diaporthe eres* CBS 160.32.

Transporter Class	*D. amygdali* CAA958	*D. eres* CBS 160.32
Channels and pores (TC 1)	348	348
Electrochemical potential-driven transporters (TC 2)	973	911
Primary active transporters (TC 3)	366	371
Group translocators (TC 4)	48	39
Transmembrane electron carriers (TC 5)	14	13
Accessory factors involved in transport (TC 8)	270	266
Incompletely characterized transport systems (TC 9)	306	290
TOTAL	2325	2238

**Table 5:** Genomic features of the *Diaporthe* species analyzed: ND, no data.

Species	Strain	Host	BUSCO * Completeness %	Genome Size (Mb)	GC Content %	Predicted Genes	Secreted Proteins	CAZymes	BGCs	GenBank Accession Number
*Diaporthe ampelina*	DA912	Grapevine	98.7	53.4	52.8	10,704	*ND*	696	105	LWAD01000000
*Diaporthe amygdali*	CAA958	Blueberry	98.3	51.5	52.1	15,818	1562	856	86	This study
*Diaporthe batatas*	CRI 302-4	Sweet potato	97.9	54.4	50.6	13,037	1224	941	91	JAHWGW000000000
*Diaporthe capsici*	GY-Z16	Walnut	98.4	57.6	51.3	14,425	1488	843	103	WNXA00000000
*Diaporthe caulivora*	D57	Soybean	97.8	57.8	52.9	18,385	1501	*ND*	*ND*	*ND*
*Diaporthe citri*	ZJUD2	Citrus	98.5	59.6	47.9	15,218	1860	847	98	JADAZQ000000000
*Diaporthe citriasiana*	ZJUD30	Citrus	99.2	52.4	52.0	13,839	1643	796	89	JADWDH000000000
*Diaporthe citrichinensis*	ZJUD34	Citrus	98.3	54.5	54.1	15,928	2043	925	110	JADAZR000000000
*Diaporthe eres* (syn. *D. vaccinii*)	CBS 160.32	Blueberry	98.4	60.8	47.6	16,499	1616	859	88	This study
*Diaporthe helianthi*	DHEL01	Sunflower	98.3	63.6	43.9	13,139	1433	764	67	MAVT02000001
*Diaporthe longicolla*	MSPL 10–6	Soybean	98.2	62.0	48.6	16,597	1535	1221	174	AYRD00000000

* BUSCO, Benchmarking Universal Single-Copy Orthologs

**Table 6:** Predicted genes encoding for CAZymes in the genomes of *Diaporthe amygdali* CAA958 and *Diaporthe eres* CBS 160.32.

Classes	Total Number of Genes		Secreted CAZymes	
	*D. amygdali*	*D. eres*	*D. amygdali*	*D. eres*
GT	107	108	3	10
GH	404	398	235	40
CBM	20	25	11	4
AA	230	225	131	26
CE	63	66	44	5
PL	33	37	30	3
TOTAL	857	859	454	88

## Data Availability

All data generated and analyzed in this study are included in this article and its supplementary information. The Whole Genome Shotgun project of *Diaporthe amygdali* and *D. eres* have been deposited in GenBank under accession numbers JAJATV000000000 and JAJATR000000000, respectively. The raw sequencing data for the *Diaporthe amygdali* genome and the assembly reported in this paper is associated with NCBI BioProject PRJNA718179, BioSample SAMN18524398, and SRA SRR14151706 within the GenBank. The data for the *D. eres* genome and the assembly is associated with NCBI BioProject PRJNA763766, BioSample SAMN21449118, and SRA SRR16214651.

## Supplementary Materials

The following are available online at https://www.mdpi.com/article/10.3390/jof8080804/s1, Tables S1 and S2: gene annotation of *Diaporthe amygdali* CAA958 and *D. eres* CBS 160.32, respectively; Table S3: predicted proteins with signal peptide; Table S4: predicted proteins with transmembrane structure; Table S5: predicted secretome; Table S6: pathogen–host interaction; Table S7: effectors candidates; Table S8: predicted cellular transporters; Tables S9 and S10: predicted CAZymes; Table S11: biosynthetic gene clusters.
